# Condensed internet-delivered prolonged exposure provided soon after trauma: A randomised pilot trial

**DOI:** 10.1016/j.invent.2020.100358

**Published:** 2020-12-10

**Authors:** Maria Bragesjö, Filip K. Arnberg, Josefin Särnholm, Klara Olofsdotter Lauri, Erik Andersson

**Affiliations:** a Department of Clinical Neuroscience, Division of Psychology, Nobels väg 9, Karolinska Institutet, 171 77 Stockholm, Sweden; b National Centre for Disaster Psychiatry, Department of Neuroscience, Psychiatry, 751 24 Uppsala, Sweden; c Stress Research Institute, Stockholm University, 106 91 Stockholm, Sweden

**Keywords:** Early intervention, Prolonged exposure, Prevention, Acute stress disorder (ASD), Post-traumatic stress disorder (PTSD), Intrusive memory, Intrusions

## Abstract

Exposure to trauma is common and may have detrimental psychological consequences. Brief exposure therapy provided early after trauma has shown encouraging results in promoting recovery. To scale up treatment availability, we developed a 3-week internet-delivered intervention comprised of four modules based on prolonged exposure (condensed internet-delivered prolonged exposure; CIPE) with therapist support. In this pilot study, we assessed the feasibility, acceptability, and preliminary efficacy of CIPE delivered within 2 months after the index event. Thirty-three participants were randomised to CIPE or a waiting list (WL). The frequency, vividness and distress of intrusive recollections or flashback memories of the traumatic event were assessed using an intrusive memory smartphone app. Symptoms of post-traumatic stress were assessed by the PTSD Symptom Checklist for DSM-5 (PCL-5). The most common index traumas in the sample were rape, interpersonal violence and life-threatening accidents. A majority of participants (82%) randomised to CIPE completed all modules, and the number of logins per participant to the Internet platform was high during the three-week intervention (*M* = 19.6, *SD* = 11.8). At post-treatment, the CIPE participants had a more favourable reduction than the WL group on the vividness and distress ratings, as well as on the PCL-5 sum score (bootstrapped *d* = 0.85; 95% CI [0.25–1.45]). Treatment effects were sustained at 6-months follow up and no severe adverse events associated with the intervention were found. CIPE seems to be a feasible and possibly efficacious early intervention after trauma. Large-scale trials are needed to assess its efficacy and long-term benefits.

## Highlights

• CIPE was piloted as an early intervention after trauma
• A majority of participants completed treatment
• CIPE was found to be a feasible and acceptable early intervention after trauma
• CIPE was preliminary efficacious in reducing symptoms of post-traumatic stress

## 1 Introduction

Approximately 70% of the population will sometime during their lifetime be exposed to a psychologically traumatic event (Koenen et al., 2017). Common adverse reactions early after exposure are intrusions, avoidance, cognitive and mood changes, and hyperarousal. If these reactions are disrupting within the first month following the event, a diagnosis of acute stress disorder (ASD) can be used (American Psychiatric Association, 2013). For approximately 5–6% of the exposed, these reactions become chronic and develop into long-term symptoms of post-traumatic stress disorder (PTSD; Koenen et al., 2017). PTSD is a condition that is both debilitating in itself and additionally linked to a host of subsequent risks such as suicide, drug and alcohol dependence, sick leave, and several somatic diseases (Kessler et al., 1995; McFarlane et al., 1994; Song et al., 2018).

Early psychological interventions that aim to reduce distress or prevent long-term reactions after trauma have shown mixed results. Critical incident stress debriefing provided within the first days after the traumatic event has in some studies indicated reversed effects for at least some individuals. The method has been criticized for medicalising normal trauma reactions and one hypothesis for the potential exacerbation of symptoms of post-traumatic stress of this treatment is due to the way trauma survivors are encouraged to talk about their experience without the opportunity to emotionally process the trauma (Rose et al., 2002). Encouragingly, three trials have shown that 5 to 6 weeks of trauma-focused cognitive behaviour therapy (CBT-T) is more efficacious in reducing trauma symptoms compared to supportive counselling for individuals suffering from ASD when it is provided within a couple of weeks after exposure (Bryant et al., 1998; Bryant et al., 2000; Sijbrandij et al., 2007). A randomised trial by Rothbaum et al. (2012) found that a condensed three-session prolonged exposure (PE) intervention significantly reduced trauma symptoms compared to assessment only when provided 12–24 h after exposure to individuals at a hospital emergency department. However, a more recent study could not replicate these results when comparing the three-session protocol to one session and assessment only (Maples-Keller et al., 2020). These null finding may be attributed to limited power and low symptom severity at baseline. Recent studies have also evaluated visuo-spatial interventions targeting intrusive memories and shown promising results (Horsch et al., 2017; Iyadurai et al., 2018).

These studies suggest that it is possible to reach and treat trauma survivors through brief psychological interventions at an early stage before long-term psychiatric conditions like PTSD develop. Primary care, where patients commonly seek their initial contact for advice or treatment of psychological or somatic conditions presents an opportunity for early identification and intervention of PTSD. Accordingly, PE has been adapted to fit the primary care setting (Prolonged Exposure for Primary Care; PE-PC) and to be delivered to early detected patients with PTSD (Calhoun et al., 2002; Kimerling and Calhoun, 1994; Schnurr et al., 2000). PE-PC is a brief treatment that comprises of four 30-minute sessions that include imaginal exposure to the trauma memory, in vivo exposure to trauma-related avoidance, and emotional processing of the traumatic memory. Several trials have shown its efficacy in reducing PTSD, depression, and symptoms of related mental disorders (Cigrang et al., 2017; Fedynich et al., 2019; Rauch et al., 2017).

One promising way to substantially increase the accessibility of treatment would be to deliver the intervention online. A recent Cochrane review that included 10 studies (*N* = 720) concluded that internet-based CBT (I-CBT) can be effective for patients with PTSD. However the quality of the evidence was rated as low due to the small number of included trials (Lewis et al., 2018). There are, to our knowledge, only one study that has tested an internet-delivered psychological intervention in the early aftermath of trauma. A trial by Mouthaan et al. (2013) did not find that an online package was superior to a waitlist control in reducing symptoms of post-traumatic stress provided as an early intervention at a hospital emergency department. Notably, 20% of the participants never used the intervention and an additional 40% used the online platform only once during the trial. One important feature of the Mouthaan et al. (2013) trial is that the authors used a non-selective recruitment strategy at a hospital emergency department, and the treatment was entirely self-guided without any therapist support. Adherence might be improved by selecting only individuals who experience psychological symptoms after the trauma and by adding therapist support to the treatment (Baumeister et al., 2014).

To summarize, the significant public health impact of exposure to trauma and PTSD highlights the need for easily disseminated interventions, delivered at an early stage after traumatic events. The aim of this study was to expand research on early digital interventions for trauma. Our research group developed a condensed Internet-delivered prolonged exposure intervention with therapist support (CIPE). In this pilot trial, our primary aim was to investigate if CIPE is a feasible and acceptable intervention in the context of early aftermath of trauma for self-recruited individuals suffering from daily intrusions from the traumatic event. In addition, we wanted to make a preliminary effect size evaluation and investigate if CIPE is efficacious in reducing symptoms of post-traumatic stress, in order to potentially inform a subsequent large-scale efficacy trial.

## 2 Material and method

### 2.1 Design

The study used a randomised controlled design comparing the intervention (CIPE) with a waiting-list (WL) control group at pre- and post-treatment and at 6-months follow up. The WL control group enabled us to calculate a preliminary estimate of the effect of CIPE as compared to the natural recovery of symptoms of post-traumatic stress. Study participants were self-referred adult individuals in Sweden who had been exposed to a potentially traumatic event in the last 2 months. Our aim was to reach the afflicted as soon after exposure as possible. A time limit of 2 months was set because natural recovery after trauma can be expected in a majority of persons within the first 3 months and we therefore wanted the participants to finish the intervention within that time frame (Bryant, 2003; Galea et al., 2002; Norris et al., 2002; Rothbaum et al., 1992). We decided to selectively include individuals who suffered from daily recurrent, involuntary and intrusive recollections of the traumatic event or flashback memories. The reason for using flashback memories from the traumatic event in this “indicated approach” (Saxena et al., 2006) is that flashback memories are a hallmark symptom of PTSD and have been shown to be a risk indicator of long-term psychiatric problems such as PTSD (Bryant et al., 2011). Intrusive memories in the first couple of days post trauma has also been associated with PTSD one year (Creamer et al., 2004) and 15 months later (Galatzer-Levy et al., 2014).

The study was registered at Clinicaltrials.gov (ID: NCT03850639) and approved by the Regional Ethical Review Board in Stockholm, Sweden (ID: 2019-02596). The study is reported in accordance to the CONSORT statement for nonpharmacological treatments.

### 2.2 Participants

The study was open for adult Swedish residents who had been exposed to a traumatic event according to criterion A for PTSD in the Diagnostic and Statistical Manual of Mental Disorders (i.e., exposed to actual or threatened death, serious injury, or sexual violence; American Psychiatric Association, 2013) in the past 2 months and who suffered from at least one daily intrusion from this event during the week following the registration to participate in the study. Exclusion criteria were: a) other serious psychiatric comorbidity as the primary concern (e.g., on-going substance dependence, untreated bipolar disorder, psychotic symptoms, severe depression, borderline personality disorder, and high suicide risk according to the Mini International Neuropsychiatric Interview (MINI; Sheehan et al., 1998); b) currently receiving CBT for trauma-related reactions; and c) on-going trauma-related threat (e.g. living with a violent spouse). Participants on psychotropic medication had to have a stable dose for 1 month prior to inclusion in the study. Excluded participants were provided with advice on how to seek regular mental health care.

### 2.3 Recruitment

**Fig. 1:**
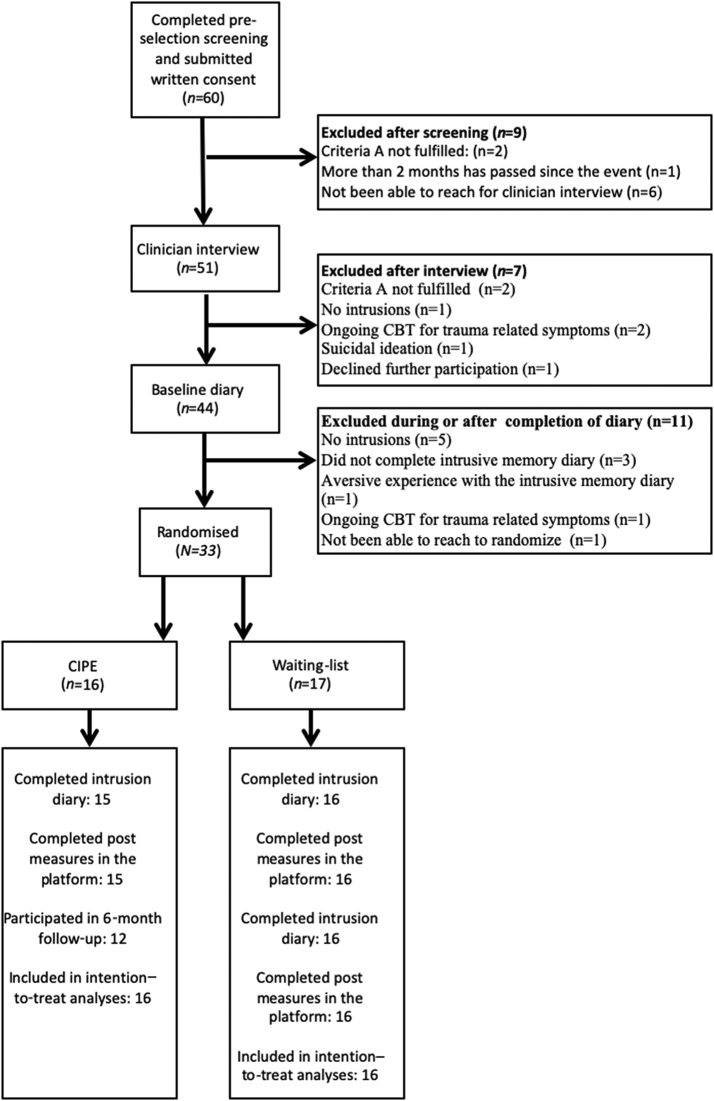
Participant flow chart.

As shown in the flowchart (Fig. 1), 65 applicants completed the pre-selection screening and provided informed consent. The most common reasons for exclusion were that the research team was not being to reach the applicant for a clinician interview (*n* = 7), no intrusive memories were present from the traumatic event (*n* = 6), and not fulfilling the criterion A for PTSD in the DSM-5 (*n* = 4). Thirty-eight participants were eligible to participate in the study. We first tested the study procedures such that five participants were allocated to the CIPE intervention immediately and went through all standard procedures in the trial. After confirming that there were no issues for these participants, we subsequently launched the pilot trial, in which we recruited 33 participants who were randomised to either CIPE (*n* = 16) or to WL (*n* = 17).

### 2.4 Baseline characteristics

The majority of participants were currently employed women in their forties with a university education (Table 1). Most participants had been exposed to more than one potentially traumatic event, both during childhood and as an adult. The most common index trauma in focus for the intervention was rape, interpersonal violence, life-threatening accident, and exposure to death. Participants were recruited from different parts of Sweden with an average time since the traumatic event of 30 days.

**Table 1:** Baseline characteristics for included participants by randomised condition.

Variable		CIPE (N = 16)	WL (N = 17)
Gender	Women	13 (81.3%)	13 (76.5%)
	Men	2 (12.5%)	4 (23.5%)
	Other	1 (6.25%)	0 (0%)
Age	Mean age (*SD*)	43 (14.21)	44 (13.15)
	Range	20–74	25–68
Highest education	Primary school	1 (6.25%)	0 (0%)
	High school	3 (18.75%)	4 (23.5%)
	College/university	8 (50%)	12 (70.6%)
	Other	4 (7%)	1 (6%)
Occupational status	Working	12 (75%)	9 (63%)
	On sick leave	3 (18.75)	3 (17.65%)
	Student	0 (0%)	1 (6%)
	Retired	1 (6.25%)	2 (12%)
	Unemployed	0 (0%)	2 (12%)
Psychiatric diagnoses according to the MINI	PTSD	5 (31.25%)	3 (17.64%)
	Current depressive episode	8 (50%)	6 (35%)
	Previous manic episodes	1 (6.25%)	1 (6%)
	Panic disorder	4 (25%)	4 (23.5%)
	Agoraphobia	3 (18.75%)	0 (0%)
	Obsessive-compulsive disorder	1 (6.25%)	0 (0%)
	Generalized anxiety disorder	1 (6.25%)	1 (6%)
Current medication	Antidepressants	1 (6.25%)	1 (6%)
	Antidepressants and atypical antipsychotics	1 (6.25%)	0 (0%)
	Antidepressants and stimulants	1 (6.25%)	0 (0%)
	Stimulants	1 (6.25%)	0 (0%)
	Sedatives/hypnotics	0 (0%)	2 (12%)
Previous trauma	In childhood	1 (6.25%)	1 (6%)
	As an adult	5 (31.25%)	2 (12%)
	Both as a child and as an adult	7 (43.75%)	9 (53%)
	None	3 (18.75%)	5 (29.41%)
Type of trauma	Rape	3 (18.75%)	1 (6%)
	Interpersonal violence	5 (31.25%)	4 (23.5%)
	Accident	3 (18.75%)	3 (17.64%)
	Witnessed death	3 (18.75%)	6 (35%)
	Traumatic birth	1 (6.25%)	1 (6%)
	Acute sickness	1 (6.25%)	1 (6%)
	Assault by animal	0 (0%)	1 (6%)
Time since trauma, days	Mean (*SD*)	30 (19.6)	36 (18.1)
	Range	1–60	2–60
Remember the traumatic event	Well	10 (62.5%)	13 (76.5%)
	Blurry	6 (37.5%)	4 (23.5%)
Degree of perceived threat (0–10). Mean (*SD*)	to death to own life	6.9 (2.8)	5 (3.9)
	to serious injury	8.1 (2.5)	5.2 (3.9)
	to death to another person's life	5.1 (3.9)	6.5 (3.9)
	to serious injury to another person	5.3 (4)	6.5 (4)
Dissociative reaction during trauma	Reports feeling of dissociation	12 (75%)	11 (65%)
Physical injury	In need of medical attention	12 (75%)	7 (41%)
	Admitted to a hospital	3 (18.75)	3 (17.65%)

CIPE = condensed internet-delivered prolonged exposure. WL = waitlist control. MINI: The Mini-International Neuropsychiatric Interview. PTSD: post-traumatic stress disorder.

### 2.5 Procedure

Participants were self-referred through advertisements in newspapers, social media, and at hospital emergency clinics throughout Sweden. Interested applicants conducted an Internet-administered screening on an encrypted webpage created for the purpose of the study to assess for eligibility. The webpage contained information about the study, data protection legislation and contact information to the principal investigator. In the registration process, a user-id and password were created for each participant. These were used with a two-factor authentication procedure to access the intervention and follow-up assessments. Participants signed informed consent before the completion of the online screening forms. Recruitment took place from 1st March to 18th of September 2019. The screening included the following questionnaires: PTSD Symptom Checklist for DSM-5 (PCL-5: Blevins et al., 2015), Montgomery Åsberg Depression Rating Scale–Self rated version (MADRS-S: Svanborg and Asberg, 1994), Alcohol Use Disorders Identification Test (AUDIT: Saunders et al., 1993), and the Drug Use Disorders Identification Test (DUDIT: Berman et al., 2005). Potentially eligible patients were subsequently assessed over the telephone by a clinical psychologist (M.B.) using the MINI 7.0.0 (Sheehan et al., 1998), a brief diagnostic interview designed to assess for 17 DSM/ICD diagnoses. In addition, information about the study was provided over the phone prior to the MINI. The interviews were typically conducted on one of the following weekdays after registration at a time slot that suited the participant. To confirm the presence of daily intrusions, the applicant completed a baseline measure assessing the daily number of intrusions over 1 week, starting at the day of the interview. After completion along with meeting the eligibility criteria, the participant was randomised to either immediate CIPE for 3 weeks or to the control WL condition for the same amount of time. The post-intervention assessment, which was the primary endpoint, included the 1-week intrusive memory diary and self-report measures on the Internet platform. Participants randomised to WL were offered the active intervention after its completion. Long-term follow-up assessments with the PCL-5, MADRS-S, and EQ-5D were also conducted at the 6-month follow up. The intrusive memory diary was omitted at the 6-month follow up in order to reduce the burden on the participants.

### 2.6 Measures

#### 2.6.1 Primary outcome measures

##### 2.6.1.1 Feasibility

Feasibility was assessed by evaluating the study procedures and provision of the intervention (Orsmond and Cohn, 2015). This was done by assessing the number of imaginal and in vivo exposure made by the participants during the intervention, number of completed modules, frequency of log-ins and messages sent to the psychologists, the participants' overall satisfaction with the intervention, and the frequency of possible negative side effects and adverse events related to the intervention.

##### 2.6.1.2 Acceptability

Intervention acceptability was defined in accordance to the definition by Sekhon et al. (2017): “*the extent to which people delivering or receiving a healthcare intervention consider it to be appropriate*, *based on anticipated or experienced cognitive and emotional responses to the intervention*” (p. 1). This definition considers acceptability to include seven component constructs: affective attitude, burden, perceived effectiveness, ethicality, intervention coherence, opportunity costs, and self-efficacy. Intervention acceptability was assessed using a brief semi-structured interview at treatment completion. The interview included questions addressing intervention satisfaction, perception of gains made from the intervention (e.g., How satisfied are you with the given intervention?) whether the participants would recommend the intervention to someone else recently exposed to trauma, opinions/experience of the intervention and its components including suggestions for improvement (e.g., Do you have any suggestions for the improvement of the intervention?). We also considered level of intervention usage, adverse events related to the intervention, and number of individuals offered the intervention but declined indicative of intervention acceptability.

##### 2.6.1.3 Secondary outcome measures

Daily occurrence of recurrent, involuntary and intrusive recollections of the traumatic event or flashback memories was assessed with a smartphone app (Trauma logbook) at baseline and post-treatment. This app was specifically developed for this current trial and has not been tested previously. It is a digital adaptation of a pen-and-paper intrusive memory diary that was developed by Holmes and colleagues (Holmes et al., 2009; Holmes et al., 2010; James et al., 2015) which has been used successfully in previous trials using trained assessors (Horsch et al., 2017; Iyadurai et al., 2018). Screenshots of the app can be found in the online supplement (eFigure 1). The participants downloaded the app from Google Play or Apple App Store and were provided with brief verbal instructions on how to use it by the assessor (details are provided in the online supplement) and were asked to make test registrations in order to understand the procedure before activating the app. The app was activated using a code provided by the assessor. The participants were asked to report the number of daily intrusive recollections of the traumatic event or flashback memories during four time periods during the day (i.e., morning, afternoon, evening, and night) and to rate the vividness of and distress from each intrusion on a 7-point scale ranging from no vividness/no distress to extremely vivid/distressing (feels like it is happening now/the most distressing experienced ever). Intrusive memory diary data from the smart phone app were accessed through a secure administrative system. Automatic reminders were sent out if the participants did not register. Participants who did not want to use the app, or in the case of app malfunction, it was also possible to complete the intrusive memory diary in a pen-and-paper format or to report the occurrence of intrusions by phone. All registrations were assessed for validity by the principal investigator after study completion but before unblinding. Each registration included a free text field on the subject of the intrusion, which the principal investigator assessed to be linked to the index traumatic event or not. The goal was to differentiate between intrusive or flashback memories related to the index event (e.g., “burning car, people screaming”) and other type or intrusive thoughts (e.g., “what if I fail my exam?”).

All self-report measures were administered via the secure Internet platform. In general, internet-delivered self-report measures have been found to generate acceptable psychometric properties (Ritter et al., 2004). We assessed the four domains of trauma symptoms in PTSD according to the DSM-5 (intrusions, avoidance, changes in cognition and mood, and hyperarousal) using the self-report questionnaire PCL-5. The PCL-5 assesses the 20 PTSD symptoms as outlined in the DSM-5 during the past month on a 4-point scale. A total symptom severity score (range 0–80) is obtained by summing the scores for each item. The PCL-5 is a reliable and valid measure with excellent internal consistency (alpha = 0.95) and good test-retest reliability (Blevins et al., 2015; Bovin et al., 2016). In the current study we adapted the Swedish PCL-5 to a shorter recall period and asked the participants to rate their symptoms during the last 7 days at post-treatment. As some participants had only a few days since the accident when included in the study, the screening/baseline recall period wording was set to “since the accident”. A cut-off of 29 has been found to be indicative of probable PTSD in a Swedish sample (Bondjers, 2020).

Depressive symptoms were assessed using the MADRS-S, which includes nine items regarding sadness, inner tension, reduced sleep, reduced appetite, concentration difficulties, lassitude, inability to feel, pessimistic thoughts, and suicidal thoughts that are rated on a scale from zero to six for a maximum score of 54 points. The MADRS-S has been shown to be sensitive to change and is therefore suitable for measuring the effect of treatment and has also shown good psychometric properties. Cronbach's alpha has for example been reported to be high and vary between 0.82 and 0.90 (Svanborg and Asberg, 1994).

Quality of life was assessed using the EQ-5D, which is a generic measure of quality of life in which health status is defined in terms of the five dimensions of mobility, self-care, usual activities, pain/discomfort, and anxiety/depression. Each dimension has three qualifying levels of responses (no problems, some problems, and unable to/extreme problems). The EQ-5D defines a total of 243 unique health states, and results can be reported either in terms of individual dimensions or as a single index score (Rabin and Charro, 2001). In this study we calculated the single index score.

The frequency and nature of any possible unwanted effects (e.g., increased symptoms or stress) associated with the intervention were assessed using a self-report questionnaire at post-treatment and at the 6-month follow up. The self-report questionnaire has been used in a previous trial with similar results as face-to-face interviews (Andersson et al., 2015). Participants reported whether they had experienced any form of adverse events that potentially could be related to participation in the study. In this self-report questionnaire, participants are asked to report any adverse events caused by the participation in the treatment. If the participant reports an event, follow-up questions are provided assessing intensity, duration, and severity. In addition, participant had the possibility to report potential adverse events during the intervention period, either through the message function in the internet platform or by contacting the study personnel by phone.

### 2.7 Condensed internet-delivered prolonged exposure (CIPE)

The CIPE intervention used in the current study is a brief PE protocol adapted to an Internet-delivered format. CIPE comprises of four text-based modules that the participant gained access to sequentially after completing homework exercises (see eFigure 4 for details in content). All material was also available in an audio-file format, providing participants with a flexible way to access the information. The participants were encouraged to have daily contact with their therapist in order to make full use of the 3-week treatment period. Participants were informed that they were expected to work with the treatment material approximately 6 h per week.

The first module included an introduction to the intervention and psychoeducation about common reactions after experiencing psychological trauma. In addition, the participants were introduced to controlled breathing as a way to deal with general stress and they were encouraged to practice controlled breathing three times a day. The second module included imaginal exposure and processing. We provided the participants with information on how to be aware of common pitfalls in imaginal exposure such as over-engagement, under-engagement, and zooming out during the revisiting of the traumatic memory (Foa et al., 2019). Case examples that illustrated these challenges and how to address them, as well as for how to conduct imaginal exposure were also provided. The participants could revisit the traumatic memory either by recording a verbal recount of the traumatic event or writing it down on paper/computer. The participants were instructed to revisit the traumatic memory for at least 20 min daily followed by 15 min of processing. Processing aims to help the participant gain a more helpful perspective on the traumatic event and challenge erroneous thought about oneself and the world. Module three expanded the imaginal exposure exercises in that the participant were instructed on how to approach the most distressing parts of the memory and how to deal with trauma hotspots (Foa et al., 2019). This module also included instructions for in vivo exposure: how to gradually approach safe or low-risk situations that participants had avoided since the traumatic event. Participants were asked to compile a list of situations and grade them according to their own subjective unit of distress scale. We provided the participants with examples of situations that are common for trauma survivors to avoid and case descriptions of how other trauma-exposed individuals have worked with in vivo exposure and what kinds of problems might arise, such as engaging in safety behaviours. Care was taken in this module to highlight to the participants that situations containing an actual threat were not to be approached. The in vivo exposure exercises were carefully planned together with the therapist using the email system in the platform.

During the module two and three, the participants were encouraged to keep daily contact with the therapist and use provided digital worksheets for imaginal exposure, processing, and in vivo exposure each time they approached the memory or a situation. The participants could easily keep track of previous exercises because they were saved in the platform. Each worksheet could be duplicated and filled out an infinite number of times. The participant as well as the therapist could thus easily follow the participant's progress. Module four included a summary of the treatment, and the participant was asked to make a relapse-prevention plan for themselves.

Participants and therapists communicated through an email system within the intervention platform, and participants could expect to receive a response from their therapist within 24 h on weekdays. The therapists were instructed to guide the participants through the treatment by answering participant questions, provide support, provide encouragement on the progress made, and provide individually tailored feedback on completed assignments and on progress and difficulties. The therapist also sent out reminders, or called the participants, if they did not log in to the platform for three days or were late with submitting homework exercises. The therapists in this trial were three clinical psychologists with experience in the treatment of PTSD (M.B., J.S. and K.O.L.). The psychologists received a three-hour training in the PE protocol used in this study. One of the therapists (M.B.) has been extensively trained by the developer of PE, Professor Foa, and is a supervisor and trainer in PE and had worked clinically with PTSD for 19 years. In addition, M.B. monitored all participants and provided supervision to the psychologists on demand.

### 2.8 Control condition

Participants randomised to the WL condition were informed that they would receive the CIPE intervention after 4 weeks and were given the telephone number to a study researcher in case of acute worsening of symptoms or suicidal ideation. The reason for having a no-treatment control group was that we wanted to investigate the effect of CIPE on initial trauma symptoms compared to the rate of natural recovery. Furthermore, effects from this trial could help us to properly power a subsequent large-scale trial.

### 2.9 Randomisation

Participants were consecutively randomised without constraints by an independent party (using www.random.org) in a 1:1 ratio. Participants were allocated to one of three different psychologists on the basis of available time slots in their calendars.

### 2.10 Statistical analyses

Statistical analyses were conducted according to the intention-to-treat principle in STATA 16.1. Group, time, and group × time interaction effects from pre- to posttreatment for all scales were estimated using a mixed effects regression framework with maximum likelihood estimations and random intercepts. The reason for using mixed effects models is due to their effectiveness in handling missing data as well as in reducing the risk of committing type I errors (Gueorguieva and Krystal, 2004). For the registrations in the intrusive memory app, we analysed the mean vividness and intrusiveness obtained from the registrations obtained at pre- and post-treatment. Count data (number of registrations in the intrusion app) was analysed using a generalized estimating equations model with a negative binomial distribution. For the PCL-5, we analysed both the sum score and the subscale scores. For the MADRS-S and EQ-5D, we analysed the sum score. In order to estimate between-group effect sizes, we used the *m*_*effectsize* command in Stata developed by Professor Matteo Bottai (publicly available using the command “net install m_effectsize, from (http://www.imm.ki.se/biostatistics/stata) replace” in Stata). This command makes an estimation of the effect sizes by dividing the estimated change score in the mixed effects regression analysis by the pooled standard deviation at pre-treatment. A total of 1000 bootstrap replications were used in order to provide a 95% confidence interval around this effect size estimate. Participants who had ≥10 point reductions on the PCL-5 were classified as responders as according to the PCL-5 scoring interpretation from the National Center for PTSD to allow for future comparisons (Weathers et al., 2013.). Logistic regression analysis was used to investigate the differences in responder rates between the groups.

## 3 Results

### 3.1 Intervention feasibility and acceptability

Thirteen out of 16 participants (82%) in the CIPE group completed all modules. None of the included participants dropped out from the intervention. The mean number of logins was almost daily during the 3-week intervention period (*M* = 19.62, *SD* = 11.8). The mean number of sent messages from the participant to the therapist was 7.67 (*SD* = 5.78) and the mean number of sent messages from the therapist to the participant was 10.2 (*SD* = 4.81). On average, each therapist spent *M* = 45 min per participant writing and reading messages/worksheets.

### 3.2 Intervention acceptability

All participants reported being ‘very satisfied’ or ‘satisfied’ with the intervention and would recommend the intervention to others. Participants perceived the intervention overall as tolerable, effective and coherent. At post-treatment, four participants reported an adverse event from participating in the study. The adverse advents described were; the intrusive memory app to be difficult to manage, mainly due to various software malfunctions (*n* = 2), the CIPE intervention to be stressful and had wished for more treatment time (*n* = 1), heart palpitations and reminders of another traumatic event during the CIPE intervention (n = 1). The were no reported adverse event at the 6-months follow-up.

The attrition rate was low for the secondary outcome measures. Eighty-eight percent of the participants in the CIPE condition completed the 1-week intrusive memory diary post-treatment, starting at the day after completion of the intervention, compared to 94% in the WL group. One participant was excluded from the intrusion registrations as the registrations were not considered intrusive memories but other types of thoughts. Ninety-four percent of the participants (both groups) logged in to the secure website and completed the post-assessment.

### 3.3 Secondary outcome data

**Fig. 2:**
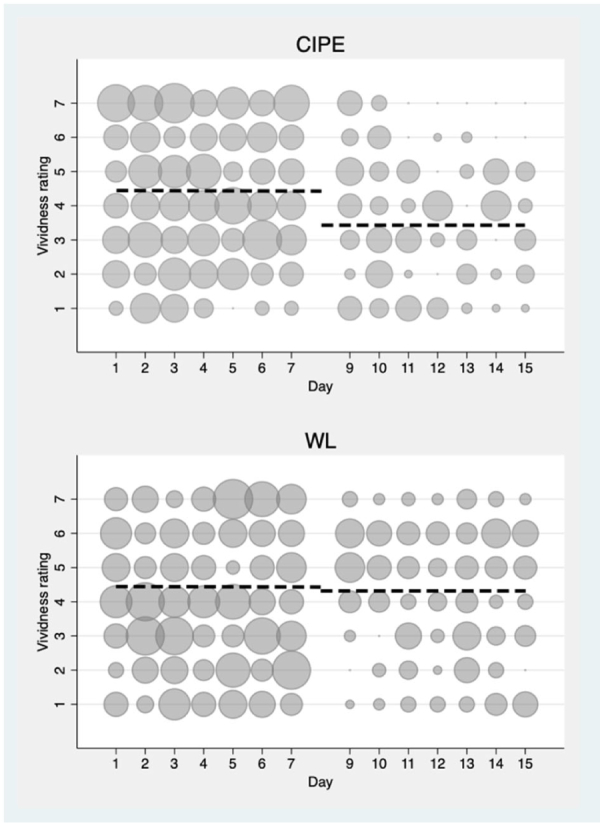
Frequency scatter graphs of daily vividness ratings associated with each intrusion recorded in the intrusive memory app or by paper and s tencil for CIPE and W'L at pre- and post-intervention. The circle size illustrates number of participants who reported the indicated rating of vividness each day.

**Fig. 3:**
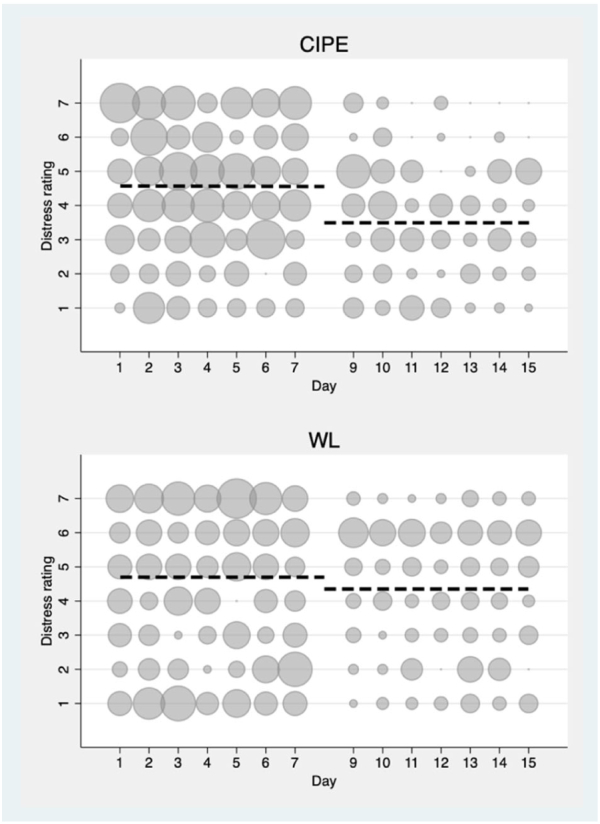
Frequency scatter graphs of daily distess ratings associated with each intrusion recorded in the intrusive memory app or by paper and stencil for CIPE and W'L at pre- and post-intervention. The circle size illustrates number of participants who reported the indicated rating of distress each day.

Participants in the CIPE group reported on average 8.66 (SE = 2.25) daily intrusive memories at pre-treatment (911 registrations), and this was reduced to 4.23 (SE = 1.13) at post-treatment (325 registrations), while the corresponding figures for the WL group were 5.96 (SE = 1.46) at pre-treatment (733 registrations) and 3.24 (SE = 0.88) at post-treatment, (330 registrations, B = −1.87, Z = −1.43, *p* = .153 [see also eFigure 2]). Participants in the CIPE group had an average vividness rating of 4.43 at pre-treatment, and this was significantly reduced to 3.42 at post-treatment (*B* = −1.01, Z = −11.21, *p* < .0001). The mean distress rating for the CIPE group was 4.55 at baseline, and this was significantly reduced to 3.49 at post-treatment (*B* = −1.06, Z = 11.51, *p* < .0001). For the participants randomised to WL, the corresponding figures for the vividness rating were 4.43 at baseline and 4.31 at post-treatment (*p* = .165), and they had a distress rating of 4.69 at baseline that was reduced to 4.33 at post-treatment (*B* = −0.36, Z = −4.55, *p* < .001). The CIPE group had significantly greater reductions in vividness (*B* = −0.90, Z = −7.34, *p* < .001) and distress (*B* = −0.70, Z = −5.74, *p* < .001) compared to the WL group. Fig. 2, Fig. 3 show detailed results for the vividness and distress ratings.

Mixed effects model analysis showed a significant interaction of group and time for the PCL-5, indicating a more favourable decrease in symptoms in the CIPE group (Table 2). Between-group effect sizes were in the large range at post-intervention (CIPE vs. WL, bootstrapped Cohen's *d* = 0.85, 95% CI [0.25–1.45]). In the CIPE group, 80% were classified as responders (≥10-point reductions on the PCL-5) and the corresponding figure in the control group was 30% (*B* = 2.17, Z = 2.59, *p* < .05). Mixed-effects model analysis showed no significant interaction of group and time for MADRS-S (*p* = .23) or EQ-5D (*p* = .63) indicating no difference in improvement between the groups for those measures.

**Table 2:** Intention-to-treat results for secondary outcomes.

	CIPE Pre-treatment		WL Pre-treatment		CIPE Post-treatment		WL Post-treatment		CIPE 6 months FU		Mixed effects model			Effect size
	*M*	*SD*	*M*	*SD*	*M*	*SD*	*M*	*SD*	*M*	*SD*	*B*	*Z*	*p*	Bootstrapped *d* (*95*% *CI*)
PCL-5 sum	52.56	13.1	47.52	13.16	30.27	15.39	37.93	15.77	11.67	9.58	12.28	3.12	0.002	0.85 (0.25–1.45)
PCL-5 intrus	13.75	4.36	12.94	3.86	7.53	4.54	10.75	4.45	3.33	3.74	4.16	2.68	0.007	0.97 (0.18–1.76)
PCL-5 avoid	5.56	2.1	5.17	12	3.00	2.39	4.43	2.00	1.58	1.50	1.79	2.07	0.038	0.84 (0.08–1.77)
PCL-5 hyper	15.5	3.50	13.58	4.43	9.26	4.72	10.68	4.61	3.08	3.28	3.28	2.42	0.015	0.75 (0.09–1.41)
PCL-5 cog	15.18	5.12	13.58	4.43	9.2	5.51	10.68	6.19	3.33	2.87	2.11	1.35	0.18	0.37 (0.20–0.94)
MADRS-S	27.88	8.54	24.29	8.57	20.33	10.27	19.63	10.17	7.67	8.39	2.78	1.19	0.23	0.30 (0.20–0.79)
EQ-5D	0.50	0.32	0.50	0.32	0.56	0.32	0.52	0.30	0.69	0.40	0.05	0.47	0.63	0.07 (−0.32–0.47)

Abbreviations: PCL-5: PTSD Symptom Checklist for DSM-5, PCL-5 sum: total sum score, PCL-5 intrus: sum score intrusion subscale, PCL-5 avoid: sum score avoidance subscale, PCL-5 hyper: sum score hyperarousal subscale, PCL-5 cog: sum score cognitions and mood subscale, EQ-5D: Euroqol-5D, MADRS-S: Montgomery Åsberg Depression Rating Scale–Self rated version.

#### 3.3.1 Crossover participants

After completing the post-assessment (1-week intrusive memory diary and self-report measures on the Internet platform), the WL participants were subsequently offered CIPE. One participant dropped out from the study, and the remaining (*N* = 16) started CIPE. Forty-four percent of those participants (*n* = 7) completed all modules. The remaining participants completed one (*n* = 8, 50%) or two modules (*n* = 1, 6%). Nine participants completed the intrusive memory diary once again and reported one intrusive memory per day on average (63 registrations). The results showed a significant within-group reduction from the start to the end of the intervention on the vividness ratings (from 4.31 to 3.56; *B* = −0.74, Z = −4.93, *p* < .001) and distress ratings (from 4.32 to 3.25; *B* = −1.04, Z = −6.54, *p* < .001) from the intrusive memory diary (details shown in eFigure 3). The mean PCL-5 sum score dropped from 37.93 (*SD* = 15.77) to 17.46 (*SD* = 11.54) points (bootstrapped within-group *d* = 1.30 95% CI [2.11–0.49]), and significant reductions were seen on all subscales (*p* < .001). Seventy percent of the crossover participants were classified as responders at their post-treatment assessment. The mean score on the MADRS-S was significantly decreased (*B* = −6.9, Z = −4.07, *p* < .001), and the mean score on the EQ-5D was significantly increased (*B* = −0.15, Z = −2.42, *p* < .05).

#### 3.3.2 6-months follow-up

Twelve participants (75%) in the CIPE group completed the 6-months follow up, at which the effects gained from the intervention were sustained. A mixed effects regression model indicated a further decline on the PCL-5 sum score (B = −9.5, Z = −1.96, *p* = .05) and on the MADRS-S (*B* = −8.4, Z = −3.06, *p* = .002) as well as sustained effects on the EQ-5D (*B* = −0.16, Z = −1.82, *p* = .07).

## 4 Discussion

This study suggests that a condensed online version of PE can be delivered shortly after a psychologically traumatic event. Engagement and adherence to the intervention was high and only a short amount of time per participant was needed for the therapists. There were only a few minor adverse events. Remote recruitment and assessment worked well, and we were able to reach individuals throughout Sweden on average 30 days since their exposure to trauma. The intervention had clinically meaningful effects on vividness and distress associated with intrusions as well as on symptoms of post-traumatic stress, although no significant interaction effect was found on the frequency of intrusions.

There are known therapist barriers in providing exposure-based interventions such as fear of symptom exacerbation and dropout (van Minnen et al., 2010), and these results might therefore be important to convey to clinicians. The results indicate feasibility and tolerability in providing imaginal exposure remotely as a brief intervention. The Internet format used in this trial might lower the threshold for individuals exposed to a psychological trauma to seek help. The low therapist time per participant might also provide a solution to the lack of suitably trained clinicians in CBT-T (Deacon and Farrell, 2013). In the face of a larger catastrophe affecting a large number of individuals, CIPE might require fewer resources than traditional face-to-face treatment. The on-going Covid-19 crisis poses a significant challenge to mental health services, and CIPE could potentially increase outreach to trauma-exposed individuals who, due to quarantine restrictions or other logistical barriers, are unable to attend face-to-face sessions.

There were sustained effects of CIPE on symptoms of post-traumatic stress and depression as well as on quality of life at the 6-months follow up. The WL control group had similar reductions in symptoms of post-traumatic stress after crossing over to the intervention. However, it should also be noted that the control group had a lower completion rate. One possible explanation for this could be study fatigue: The crossover participants had to register both daily intrusive memories for one week and conduct assessments twice before gaining access to the intervention. Some participants in the crossover group also experienced technical difficulties on the app, which could have had a negative impact on adherence to the intervention.

The trial by Mouthaan et al. (2013) found that an unguided online CBT package was not superior to WL control in reducing trauma symptoms. In contrast to their study, we found high adherence rates and a low degree of data attrition. We suggest two plausible explanations for this difference. First, the treatment used in Mouthaan et al. (2013) was completely self-guided, which is in contrast with CIPE, in which there was frequent contact between therapists and participants during the intervention period. When delivering the intervention rationale, we stressed the importance of making use of the 3-week intervention period and encouraged the participants to work at least 6 h a week with the material, to send in the worksheets each time an exercise was finished, and to have daily contact with the therapist. This quite intensive therapist-guided approach implemented in CIPE could have made a significant impact on compliance to the intervention. Previous research on Internet-delivered CBT has shown that therapist-guided protocols have more positive results than unguided ones (Baumeister et al., 2014). Second, the trial by Mouthaan et al. (2013) was conducted at an hospital emergency clinic recruiting everyone exposed to a potentially traumatic event, irrespective of the severity of their trauma-related reactions. In contrast to this universal approach to recruitment, the current CIPE study used an indicated approach (Saxena et al., 2006) in which only self-referred individuals experiencing daily intrusions from a traumatic event were included. This approach could have led to selection of more motivated participants as compared to those recruited in an hospital setting as in the Mouthaan et al. (2013) trial.

At the end of the CIPE intervention, the mean total score of PCL-5 in the CIPE group was 30. Although this was significantly lower than in the control group, a sum score of 30 indicates that some participants had high levels of posttraumatic stress. One suggestion for future research would be to investigate whether these individuals would benefit from a longer intervention period, additional treatment components, or other types of support. A qualitative analysis of the participants' experiences from the CIPE intervention could also provide important participant perspectives on this important issue. Future studies could also benefit from longer experimental control follow-up times for controlled assessments of the intervention effect on long-term changes in symptoms of posttraumatic stress. Another important future direction is to investigate different indicators to target people who would benefit from early interventions. To our knowledge, at this time no reliable algorithms are available that can distinguish individuals who after exposure to a PTE will develop PTSD with certainty. Severity and frequency of symptoms of ASD and post-traumatic stress may be one indicator worthy of pursuit.

As for reductions on specific subscales, CIPE specifically aims to reduce avoidance and facilitate emotional processing of the traumatic memory; it is therefore interesting that the largest effect sizes in this pilot trial were on the avoidance and intrusions subscales on the PCL-5. The effect size for the cognitions and mood subscale was in the lower range, which is somewhat in contrast to findings that negative cognitions may play a key role in the treatment of PTSD and mediate the decrease in symptoms of post-traumatic stress (Foa and McLean, 2016; Kumpula et al., 2017; McLean et al., 2015; Zalta et al., 2014). Although this study could not draw firm conclusions about this issue, the results raises interesting questions about whether the mechanisms in psychological treatment of trauma are different when implemented early after the traumatic event. Future research could investigate this further.

This study has several limitations that should be taken into consideration when interpreting its results. The sample size was small and included self-selected participants: it is unclear if the results can be generalized to a wider population of trauma-exposed individuals. The small sample size also reduces the power of the study and increase the margin of error, leading to uncertain estimates that need to be determined fully in larger trials. We used self-administered outcome measures, and future trials should corroborate the results using clinician-assessed instruments. In addition, the intrusion diary was provided remotely without any continuous feedback. Together with some software malfunctions that occurred, especially at the beginning of the study, this may have affected the number and accuracy of the reports on intrusive memories. However, our screening of the reports might have mitigated some types of erroneous reporting.

## 5 Conclusions

We believe that CIPE holds promise as an early intervention after a traumatic event. This study is the first to show that condensed PE is feasible and possibly efficacious to conduct in an online format early after trauma, making it an easily accessible and scalable intervention. Nonetheless, further research is warranted to assess the efficacy and long-term benefits of this intervention and a larger randomised efficacy trial is currently ongoing.

## CRediT authorship contribution statement

E.A, F.K.A., and M.B. contributed to the study design. M.B. conducted the data collection under supervision from E.A. J.S and K.O.L. assisted in data collection and contributed in writing the article. Data analysis and interpretation were done by M.B. and E.A., and they also wrote the first draft of the article. F.K.A. contributed to writing the article. All authors have read and approved the final manuscript.

## Ethics approval and consent to participate

This study was registered at Clinicaltrials.gov (ID: NCT03850639) and approved by the Regional Ethical Review Board in Stockholm, Sweden (ID: 2019-02596). Eligible patients received both written and verbal information about the study and were not included in the study until after signing informed consent.

## Consent for publication

Not applicable.

## Availability of data and material

The data supporting the conclusions of this article will be made available by the authors upon reasonable request given that the request comply with Swedish and EU laws regulating protection of identifiable data.

## Funding

The study was funded through the 10.13039/501100004359Swedish Research Council (grant 2016-02359), the 10.13039/501100007687Swedish Society of Medicine (grant 658811), and 10.13039/501100004348Stockholm County Council (grant 20170018). None of the funding organisations had any role in the conception of the study design or in the collection, analysis, or interpretation of the data, in the writing of the report, or in the decision to submit the paper.

## Declaration of competing interest

F.K.A receives royalties from Natur och Kultur for the Swedish translation of the prolonged exposure treatment manual. None of the other author report any competing interests.

## Appendix A Supplementary data

Supplementary material Image 1
