# Supplementary material for: Condensed internet-delivered prolonged exposure provided soon after trauma: A randomised pilot trial
Source: Internet Interv. 2020 Dec 10;23:100358. doi: 10.1016/j.invent.2020.100358 (PMC7771112; doi:10.1016/j.invent.2020.100358)
Supplement: Supplementary file 1 — Supplementary material [file mmc1.docx]

**Online supplement**

## Measures

Before activating the Trauma logbook app we made sure that the participants understood the concepts of intrusions and we did a thorough explanation of the phenomenon. Participants were also asked to provide examples of intrusions they had the last week and was asked to do a couple of test registrations before the app was activated and it started to save registrations. If a participant did not seem to understand the concept of intrusions, this test procedure was repeated. Screenshots from the app are shown below to illustrate the visual rating aid.


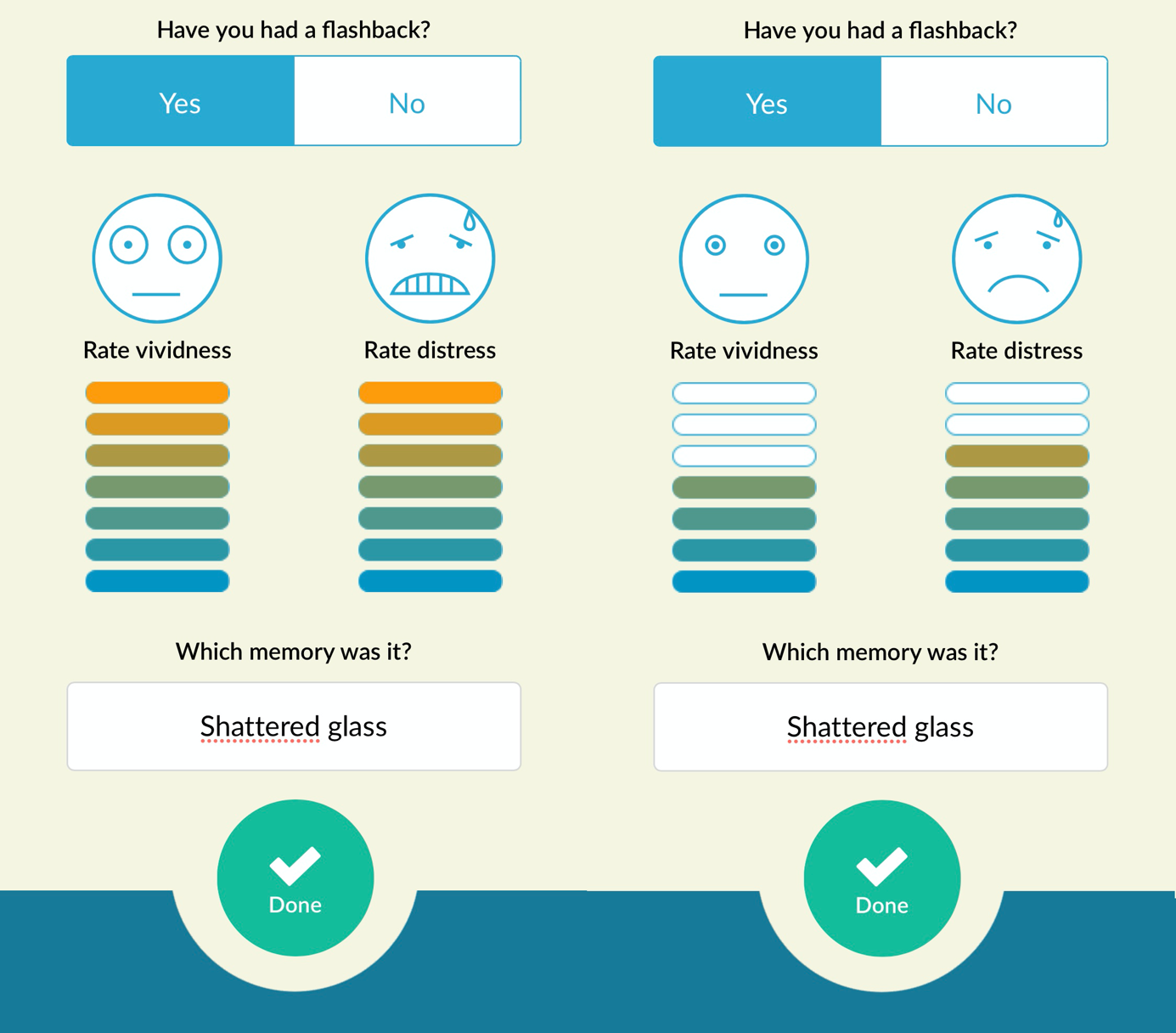


eFigure 1: *Screenshots of the intrusive memory app showing the visual aid for reporting vividness and distress*

**Secondary outcome data**


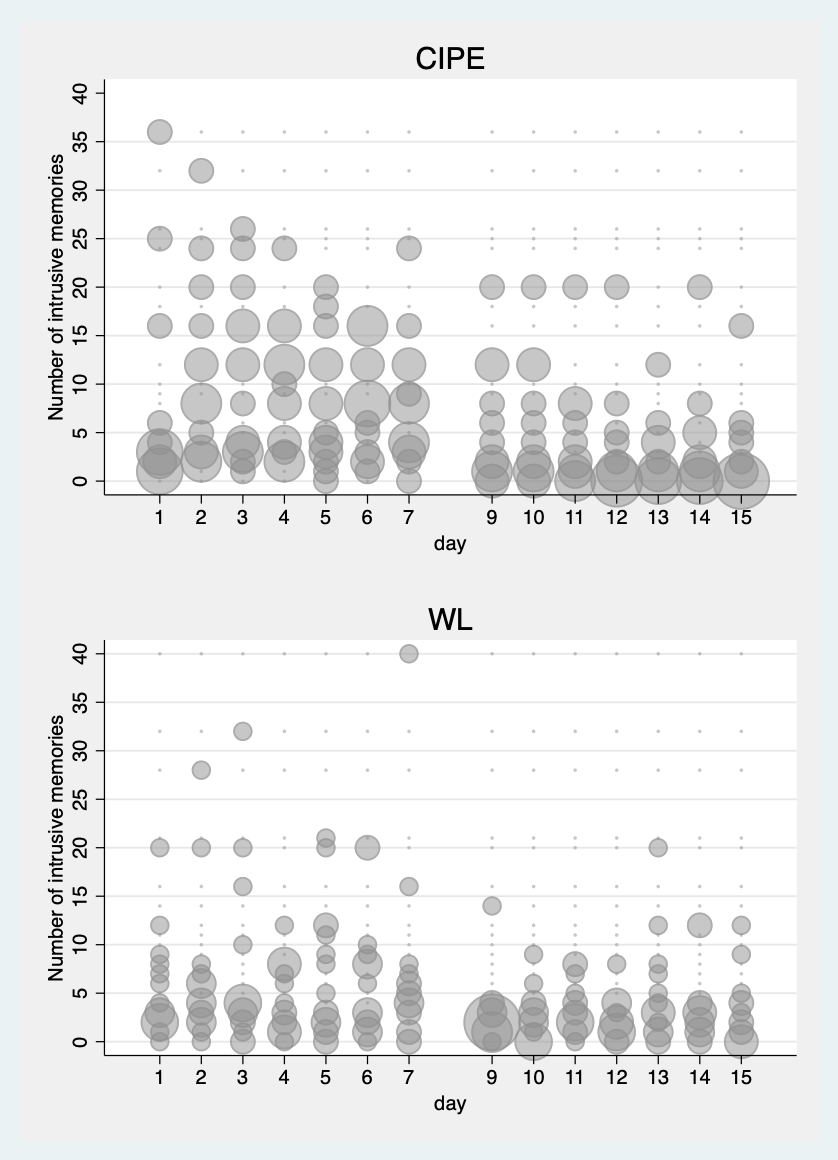


Efigure 2. *Frequency scatter graphs of daily number of intrusions recorded in the intrusive memory app or by paper and stencil for the CIPE and WL group respectively at pre- and postintervention The circle size illustrates number of participants who reported the indicated number of daily intrusions*


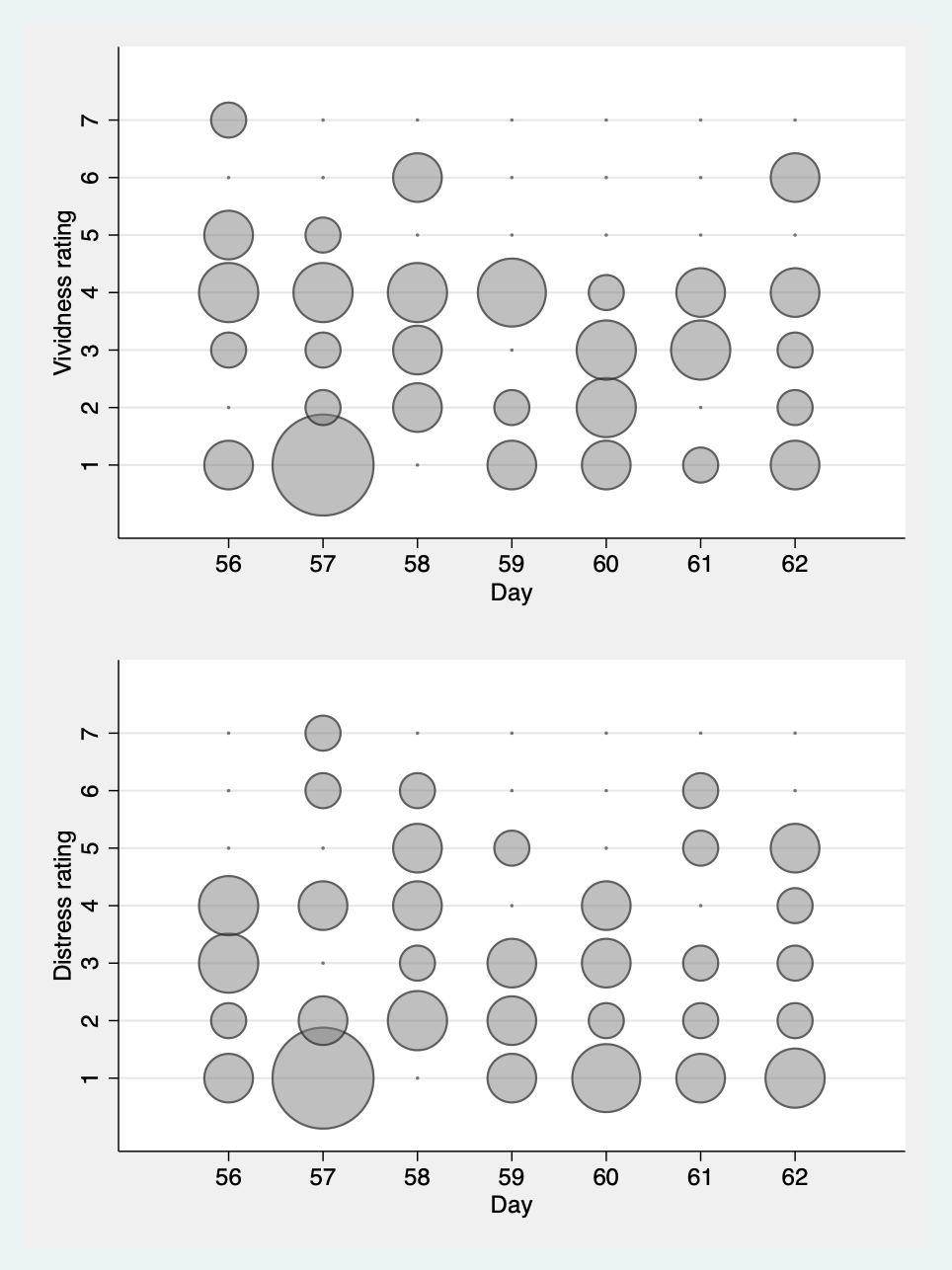


Efigure 3. *Frequency scatter graphs of daily vividness and distress ratings associated with each intrusion recorded in the intrusive memory app or by paper and stencil for WL after crossing over to treatment The circle size illustrates number of participants who reported the indicated rating of vividness and distress each day*

## Condensed internet-delivered prolonged exposure (CIPE)

|  | Content | Homework |
| --- | --- | --- |
| Module 1 | Introduction to the internet platform  Psychoeducation about common reactions to trauma  Overall rationale for the intervention  Case vignettes  Instruction for controlled breathing | Quiz  Practice controlled breathing |
| Module 2 | Rationale and instructions for imaginal exposure and processing  Common pitfalls for imaginal exposure  Case vignettes | Quiz  Practice controlled breathing  Practice imaginal exposure daily followed by processing |
| Module 3 | Rationale and instructions for imaginal exposure using hot spots  Rationale and instructions for exposure in vivo and how to construct a in vivo hierarchy  Common pitfalls for exposure in vivo  Case vignettes | Quiz  Practice controlled breathing  Practice imaginal exposure of the hot spot daily followed by processing  Practice exposure in vivo according to a predetermined hierarchy |
| Module 4 | Summary of gains  Case vignettes  Maintenance program | Create a maintenance program  Practice controlled breathing  Practice imaginal exposure of the hot spot daily  Practice exposure in vivo according to a predetermined hierarchy |

Efigure 4. *An overview of the treatment content for the four modules in CIPE*
